# State of Evidence on Oral Health Problems in Diabetic Patients: A Critical Review of the Literature

**DOI:** 10.3390/jcm10225383

**Published:** 2021-11-18

**Authors:** Miguel Ángel González-Moles, Pablo Ramos-García

**Affiliations:** 1 School of Dentistry, University of Granada, 18010 Granada, Spain; pramos@correo.ugr.es; 2 Instituto de Investigación Biosanitaria ibs.GRANADA, 18012 Granada, Spain

**Keywords:** diabetes mellitus, oral health, oral medicine, oral pathology, periodontitis, dental caries, oral cancer

## Abstract

Diabetes mellitus (DM) is a global health problem, having recognized that in the next 20 years the number of diabetic patients in the world will increase to 642 million. DM exerts enormous repercussions on general health diabetic (especially derived from vascular, cardiac, renal, ocular, or neurological affectation). It entails in addition a high number of deaths directly related to the disease, as well as a high health care cost, estimated at $673 billion annually. Oral cavity is found among all the organs and systems affected in the course of DM. Important pathologies are developed with higher prevalence, such as periodontitis (PD), alterations in salivary flow, fungal infections, oral cancer, and oral potentially malignant disorders (OPMD). It has been proven that PD hinders the metabolic control of DM and that the presence of PD increases the possibility for developing diabetes. Despite the relevance of these oral pathologies, the knowledge of primary care physicians and diabetes specialists about the importance of oral health in diabetics, as well as the knowledge of dentists about the importance of DM for oral health of patients is scarce or non-existent. It is accepted that the correct management of diabetic patients requires interdisciplinary teams, including dentists. In this critical review, the existing knowledge and evidence-degree on the preventive, clinical, diagnosis, prognosis, and therapeutic aspects of oral diseases that occur with a significant frequency in the diabetic population are developed in extension.

## 1. Introduction

Diabetes mellitus (DM) is a health problem of global importance that affects a large number of patients around the world. According to data reported by relevant international organizations (http://diabetesatlas.org/es/sections/worldwide-toll-of-diabetes.html, accessed on 15 December 2020), in the next 20 years, the number of worldwide diabetic patients will increase to 642 million people. DM exerts enormous repercussions on general health diabetic (especially derived from vascular, cardiac, renal, ocular, or neurological affectation). It entails in addition a high number of deaths directly related to the disease, as well as a high health care cost, estimated at $673 billion annually. Current scientific evidence indicates that DM is the consequence of an interaction of environmental, epigenetic, and genetic factors [1]. Among the environmental factors are fundamentally infections and the microbiota involved—of particular importance is the microbiota affecting the oral and intestinal cavities—diet and others. Epigenetics is currently considered as the link between the environment and genetics, altering gene and protein expression. Epigenetic factors—including DNA methylation, histone modification, and microRNAs (e.g., miR-15b, miR-29, or miR-122 [2])—regulate gene expression. These key events are implicated in autoimmunity and in the vulnerability of beta cells of pancreatic islets. Finally, genetic factors promote a special susceptibility to the development of the disease [3]. In this aspect, more than 60 genes, altered chromosomal loci and polymorphisms (e.g., rs12255372 and rs7903146 variants of *TCF7L2* [4]), have been implicated. Type 1 diabetes mellitus (DM1) responds to a multifactorial pathogenesis essentially linked to an autoimmune aggression mediated by autoantibodies that generates a progressive loss of insulin-producing β-cells in the pancreas [5]. On the other hand, the pathogenesis of type 2 diabetes mellitus (DM2) is essentially linked to the development of a state of resistance to the actions of insulin [6].

Oral cavity is found among the organs and systems affected in the course of DM (Table 1). Nevertheless, the information on many of the diabetic related oral diseases—with regard to diagnosis, treatment, and prevention—is limited among health care providers in diabetic patients, especially endocrinologists and family doctors. Likewise, the knowledge that dentists have on the relationships between oral health and DM, and the information on the implications of the dentist in the control of diabetic patients seem limited. Furthermore, knowledge about the aforementioned aspects is frequently based on scant scientific evidence. Undoubtedly, the prevention and control of oral pathology in diabetic patients improves their quality of life and most likely facilitates the long-term control of DM and consequently improves their prognosis. The health care providers for the treatment and follow-up of diabetic patients should be well informed of oral pathologies frequently associated with DM in order to be able to prevent, diagnose, and treat them, or if necessary, refer patients to specialized centers for their management. This most likely requires the implementation of educational programs that convey evidence-based information. In this critical review, the existing knowledge and its degree of evidence, the preventive, clinical, diagnosis, prognosis, and therapeutic aspects of oral diseases occurring with a significant higher frequency in the diabetic population are developed in extension, attending to both type 1 and type 2 DM.

## 2. Scientific Framework

We chose the critical narrative review design as the scientific framework of this paper on the basis that this type of design covers a wide range of aspects in a given topic. Furthermore, this study design offer the reader global information on a health problem with different facets (not easily achievable through other designs such as systematic reviews and meta-analyses). In this paper, we follow the concept of critical review used by Grant and Booth [27]. A critical review aims to demonstrate that the writer has extensively researched the literature and critically assessed its quality. It goes beyond merely describing the articles identified and includes some degree of analysis and conceptual innovation. An effective critical review presents, analyzes, and synthesizes material from a variety of sources. This concept is widely accepted in the international literature as evidenced by the high number of citations their work has received (5198 citations to date). The main strength of a critical review is based on offering an opportunity to “take stock” and evaluate what is the previous body of a health problem while at the same time making it possible to contribute the authors’ own opinions and experiences.

We searched MEDLINE through PubMed (as main electronic database) and Web of Science (for bibliometric analysis purposes) for studies published before the year 2021 (upper limit), with no lower date limit. Search strategy was conducted by combining thesaurus terms used by the databases (i.e., MeSH) with free terms, constructed to maximize sensitivity. In a first line general search, the root keywords and synonyms combined were “diabetes mellitus”, “oral health”, “periodontal diseases”, “oral candidiasis”, “oral cancer”, “oral potentially malignant disorders”, “caries”, “burning mouth syndrome”, and “salivary secretion alterations”. In addition, several more specific searches were conducted by combining relevant aspects of the goals to be reviewed (i.e., relationships between oral diseases and diabetes mellitus, prevention, diagnosis, prognosis, and therapeutic implications). We also manually screened the reference lists of the handled studies for additional relevant studies. Most of the revised studies were included or excluded according to an exhaustive analysis of the title, abstract, year of publication, impact of the journal, and number of citations received. Although these last two criteria may introduce a potential selection bias, its application is necessary when handling a large number of records (e.g., in the first line general search context, simply using the following syntax: (“Diabetes Mellitus”[mh] OR “type 1 diabetes”[all] OR “T1DM”[all] OR “type 2 diabetes”[all] OR “T2DM”[all] OR “diabetes”[all]) AND (“Oral Health”[mh] OR “oral health”[all] OR “mouth diseases”[all] OR “Periodontitis”[mh] OR “periodontitis”[all] OR “periodontal diseases”[all] OR “Mouth Neoplasms”[mh] OR “Mouth Neoplasms”[all] OR “oral squamous cell carcinoma”[all] OR “oral cancer”[all] OR “oral potentially malignant disorders”[all] OR “OPMD”[all] OR (“oral”[all] AND precancer*[all]) OR “Leukoplakia, Oral”[mh] OR “leukoplakia”[all] OR “erythroplakia”[all] OR “Lichen Planus, Oral”[mh] OR “oral lichen planus”[all] OR “Oral Submucous Fibrosis”[mh] OR “oral submucous fibrosis”[all] OR “Dental Caries”[mh] OR “caries”[all] OR “carious”[all] OR “dental decay”[all] OR “Burning Mouth Syndrome”[mh] OR “Burning Mouth Syndrome”[all] OR “BMS”[all] OR “Salivary Gland Diseases”[mh] OR “xerostomia”[mh] OR “xerostomia“[all] OR “dry mouth”[all] OR “hyposalivation”[all]), more than 7000 registers were retrieved). We would also like to clarify that a potential selection bias would only affect to the identification of primary-level studies. Given our effort to develop this review from an evidence-based scientific context, we applied optimal search filters designed for retrieving systematic reviews and meta-analyses (i.e., *Centre for Reviews and Dissemination-CRD* filter; sensitivity = 99.5%, 95%CI = 97.3–99.9 [28,29]). This approach should overcome the potential selection bias, decreasing the rate of missing systematic reviews.

## 3. Periodontitis

The concept of periodontitis (PD)—according to the new classification scheme for periodontal and peri-implant diseases and conditions [7]—is characterized by microbially-associated, host-mediated inflammation that results in loss of periodontal attachment [8]. PD disease drives the activation of host-derived proteinases with loss of marginal periodontal ligament fibers, apical migration of the junctional epithelium, and apical spread of the bacterial biofilm along the root surface of teeth [8]. Initially, bacterial biofilm formation begins gingival inflammation (i.e., dental-biofilm induced gingivitis [7]); nevertheless, PD initiation and progression is dependent on dysbiotic ecological changes in the microbiome. It occurs in response to nutrients from gingival inflammation and tissue breakdown products with the enrichment of some species and anti-bacterial mechanisms that attempt to contain the microorganisms within the gingival sulcus area once inflammation has initiated [8]. Furthermore, a multifactorial origin influenced by additional risk factors, such as smoking, is now supported on the immunoinflammatory response that trigger the dysbiotic microbiome changes, and also likely influence severity of PD for such individuals [8]. PD is an important health problem because of its prevalence and the systemic repercussions that it entails. Epidemiological studies have reported that 10–15% of the worldwide population suffers from advanced PD [30]. Likewise, the association between PD and some systemic disorders including cardiovascular and metabolic diseases is well known [31,32,33].

DM is the most prevalent systemic disease in which it has been shown, after extensive research, that it predisposes to the development of PD [34,35,36,37]. A recent meta-analysis [38] that collected information from 27 studies (3092 diabetic patients and 23,494 controls) has reported a prevalence of PD of 67.8% in patients with DM and 35.5% in controls (odds ratio [OR] = 1.85; 95%CI = 1.61–2.11), results that in an unappealable way give an idea of the magnitude of the problem. Furthermore, cohorts that include patients with DM1 and DM2 report a higher prevalence of PD in DM1 (78.8% compared to DM2 (70.5%); OR = 2.60 vs. OR = 1.71). A recent systematic review and meta-analysis [39] has also confirmed that DM1 is a relevant risk factor for the development of PD, with a proportion of patients affected more than double for DM1 compared to non-diabetic individuals. In addition, another recent systematic review [40] has also reported an evident bidirectional epidemiological relationship between DM2 and PD, such that the prevalence of DM2 was significantly higher in patients with PD (OR = 4.04, *p* < 0.001), and vice versa (OR = 1.58, *p* < 0.001). The association of DM and PD has recently been considered a comorbidity [41,42].

### 3.1. Mechanisms Linking DM and PD

**Figure 1:**
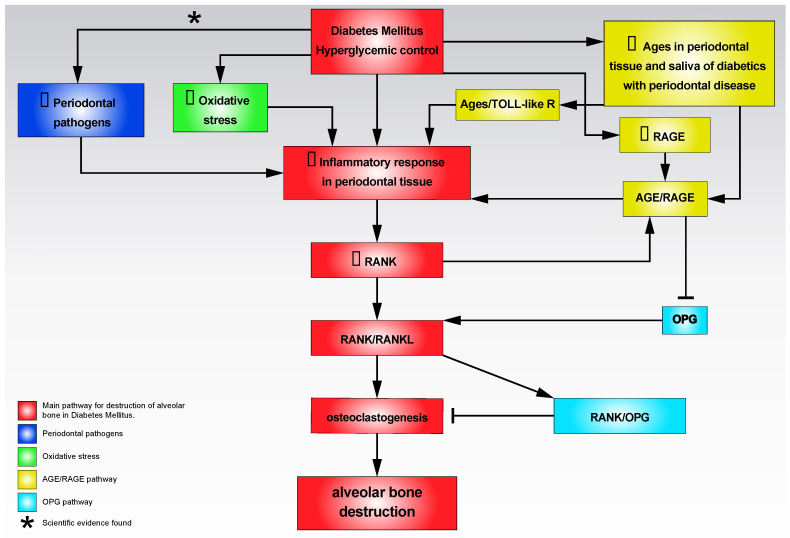
Diabetes Mellitus with a poor control generates sustained hyperglycemia, which in turn induces an increase in the inflammatory response in the periodontal tissue; this in turn stimulates the RANK/RANKL axis with an increase in osteoclastogenesis and destruction of the alveolar bone, which will conclude with the loss of teeth—one of the hallmarks in periodontitis. The increase in the number of periodontal pathogens, the increase in reactive oxygen species (ROS), and the increase in the expression of role of advanced glycation end products (AGE) and its receptor (RAGE) also activate the inflammatory response in the periodontal tissue. The receptor activator of nuclear factor κB (RANK)/RANK-Ligand (RANKL)-RANK/osteoprotegerin (OPG) balance will be important in maintaining periodontal bone homeostasis.

Poorly controlled DM generates sustained hyperglycemia, which in turn induces an increase in the inflammatory response in the periodontal tissue; this stimulates the receptor activator of nuclear factor κB (RANK)/RANK-Ligand (RANKL) axis with an increase in osteoclastogenesis and destruction of the alveolar bone, which will conclude with the clinical attachment loss, one of the PD hallmarks. The existing scientific evidence on the biological mechanisms linking DM and PD is detailed below (Figure 1).

#### 3.1.1. Impact of DM on the Oral Microbiota

It should be recognized that there is very limited and contradictory scientific evidence on the possible impact that DM may exert on the oral microbiota [41,43]. A narrative review [44] has reported that DM1 and DM2 do not have a significant effect on the composition of the periodontal microbiota and that glycemic control level does not seem to significantly influence the composition of the subgingival biofilm. On the contrary, some studies [45,46,47,48] indicate that in patients with DM, poor glycemic control could translate into a high number of periodontal pathogens. Currently, we know that these periodontal pathogens are related to the onset and exacerbation of PD [49]. However, the main limitation presented by the evidence on this subject is related to most of these studies are cross-sectional. This study design makes difficult to determine if the more than frequent concomitance of PD and DM responds to a causal relationship or is the result of the presence of common risk factors [41].

#### 3.1.2. Pro-Inflammatory Mediators in Patients with PD and DM

It is currently known that the penetration of periodontal pathogens into the periodontal connective tissue triggers an inflammatory response linked to the development and progression of PD [50]. Evidence from clinical studies supports that DM with poor glycemic control is associated with significantly high levels of pro-inflammatory mediators in gingival tissue [43]. The pathway that best documents the comorbidity between DM with poor glycemic control and PD is inflammation, having shown that an evident local and systemic inflammatory process underlies both conditions that determines its evolution and severity [41]. In vitro and in vivo studies in humans strongly indicate that DM is associated, in a proportional way to glycemic control, with a higher expression of pro-inflammatory mediators in periodontal tissue (TNF-α, IL-6, -8, -10, -12, α1β, substance P, eotaxin, macrophage inflammatory protein 1a, GM-CSF, MMP-1, ICAM1, RANKL, PGE2, Toll-like receptor-2, -4, and -9, caspase 3) and with the activation of the Th-17 pathway [44,51,52,53,54,55,56,57,58,59,60,61,62,63,64,65,66,67]. These observations have also been reported in animal models that have evidenced a significantly greater inflammatory response in diabetic vs. non-diabetic, having suggested that periodontal bacteria induce the upregulation of several pro-inflammatory and pro-apoptotic genes in diabetes [68,69].

It has also been pointed out that hyperglycemia and the conditions associated with DM can promote oxidative stress [70] through different pathways with the consequent influence on the inflammatory response. Reactive oxygen species (ROS) has been reported to stimulate the production of pro-inflammatory cytokines through the activation of MAPK, NF-Kβ, Wnt, NALP3 inflammosome pathways, and the activation of the transcription factor FoxO [71,72,73,74].

#### 3.1.3. Role of Advanced Glycation End Products (AGE) and Its Receptor (RAGE) in the Development of PD in Diabetes

An important effect of chronic hyperglycemia in uncontrolled DM is related to the non-enzymatic glycation of proteins and lipids, which results in the formation of AGEs. Higher levels of AGEs have been reported in the serum of patients with DM2 in relation to the extent of their PD [75]. The accumulation of AGEs can lead to cellular stress exerting pro-inflammatory and oxidative effects directly or through their interaction with RAGEs. RAGE is a multiligand receptor belonging to the immunoglobulin superfamily of cell-surface molecules [76] that is overexpressed in DM and has been shown to play a role in the development and progression of some complications of diabetes [77] and also in PD in these patients. In this sense, in diabetic mice it has been shown that the loss of bone linked to the infection by *Porphyromonas gingivalis* was mediated by the overexpression of AGE and RAGE [44]. It has also been reported that RAGE contributes to impaired tissue repair in surgical wounds in a diabetic mouse model, and that inhibition of RAGE-mediated signaling increased the rate of tissue healing and repair [78]. Likewise, it has been shown that the AGE-RAGE interaction delays bone healing in the absence of infection, both in osteoblast cultures and in craniotomies in animal models [79]. Finally, AGE could also bind to Toll-like receptors [44]. A significant increase in the expression of these receptors has been observed in the gingival tissue of patients with DM and PD [80], having reported that their activation exerts a pro-inflammatory effect in diabetics similar to that displayed by RAGE, which is especially significant for the Toll-like receptor 4 [81]. Through this pathway, the AGE-Toll-like receptors interaction can increase inflammation and tissue destruction in diabetic PD.

#### 3.1.4. Role of Hyperglycemia in Bone Destruction in PD

The final biological event with the greatest clinical implications in PD is tissue destruction, including destruction of the alveolar bone with consequent tooth loss. The destruction of the alveolar bone is essentially due to the stimulus of the RANK for its ligand (RANKL). RANK is mainly expressed in the membrane of osteoclasts and preosteoclasts and binds to RANKL which is secreted by T cells, indicating that the inflammation inherent in PD induces destruction of the alveolar bone through the stimulation of osteoclastogenesis related to the pathway RANK/RANKL [41]. The natural antagonist of RANKL is osteoprotegerin (OPG), in such a way that the RANK/OPG binding induces the inhibition of osteoclastogenesis. The RANK/OPG ratio is therefore a determining factor in the metabolism and homeostasis of the alveolar bone [41]. Several studies have indicated that DM with poor glycemic control favors the destruction of the alveolar bone in patients with PD mediated by the activation of the RANK/RANKL axis [82,83,84,85]. Increased levels of RANKL have been reported in periodontal tissue and crevicular fluid from diabetic patients with poor glycemic control [44,86,87], as well as increased levels of soluble RANKL [82,88] and an increase in the RANK/OPG ratio in poorly controlled DM [82,83]. Studies on animal experimentation also indicate an increase in osteoclastic activity linked to an increase in RANKL levels [89,90,91,92]. Finally, it has been interestingly pointed out that the AGE/RAGE axis can also contribute to osteoclastogenesis via increased expression of RANKL and downregulation of OPG in various cell types [93,94]. In an animal model, an increase in osteoclastic activity linked to overexpression of AGEs has been reported, while animals lacking RAGEs exhibited an increase in bone mass and a decrease in the number of osteoclasts [94,95].

### 3.2. DM Increases the Severity of PD

The existing evidence in this regard indicates that patients with DM are at greater risk of developing more severe PD [96,97,98,99,100,101,102]. The parameters most commonly used to measure the severity of PD are the probing depth or pocket depth, the bacterial plaque index, the level of clinical anchorage, which constitutes an important indicator of tissue damage, the number of missing teeth, and the rate of bleeding on probing. A systematic review and meta-analysis [38] has indicated that all these severity indicators are significantly more altered in DM compared to controls. Probing depth was significantly deeper in diabetics compared with controls (mean difference [MD] = 0.23 mm, 95% CI = 0.17–0.29, *p* < 0.001) [38]; plaque index was significantly elevated in the diabetic group (MD = 0.20 mm, 95% CI = 0.18–0.23, *p* < 0.001) [38]; clinical attachment level also reflected higher degree of damage to periodontal tissue in diabetics (MD = 0.39 mm; 95% CI = 0.28–0.50, *p* < 0.001) [38]; diabetics with periodontitis had on average less teeth than the non-diabetic group with periodontitis (MD = −2.14 teeth, 95% CI= −2.87 to −1.40, *p* < 0.001) [38]; bleeding on probing was found affecting more teeth in the diabetic group compared with the control group (MD = 7.90 teeth; 95% CI, 4.24–1.56, *p* < 0.001) [38]. In summary, this systematic review shows with the higher quality of evidence to date that severity of periodontitis is greater in patients with diabetes than in non-diabetic populations. This is relevant for clinical practice and confirms that oral cavity assessment should form a routine part in the clinical evaluation of patients with DM [38].

### 3.3. PD Worsens the Control and Prognosis of DM

Several studies provide evidence on the negative effect that PD has on the prognosis of diabetes both in terms of mortality and the appearance of DM typical complications [103,104,105,106,107,108]. A study carried out in Pima Indians—an ethnic group that lives in the state of Arizona (USA) and in the states of Sonora and Chihuahua (Mexico) that shows a high prevalence of DM—reported a significant increase in mortality adjusted for sex and age directly related to the control of their PD. Thus, in diabetic patients without PD or with PD with good control, mortality was 3.7 deaths/1000 inhabitants/year, while in diabetic patients with poor control of PD, mortality amounted to 28.4 deaths/1000 inhabitants/year [103]. Likewise, a large study [104] has reported an increase in cardiovascular mortality in diabetic patients with PD and chronic kidney disease.

Diabetic patients with PD also have a higher risk of complications typical of DM [105,106]; it was published after a joint consensus meeting between the International Diabetes Federation and the European Federation of Periodontology [43], derived from the analysis of 14 studies that included 31,988 patients, so diabetic retinopathy is significantly associated with PD (OR = 1.2–2.8) and the severity of PD is correlated with the severity of retinopathy. Likewise, in patients with DM1 and DM2 with PD there is a higher frequency of kidney complications. Furthermore, a significant association was also reported between DM with PD and the risk of neuropathic foot ulcers development (OR = 6.6); finally, the risk of cardiovascular complications (coronary heart disease, cerebrovascular events and subclinical heart disease) is also significantly increased in diabetic patients with PD.

There is sufficient evidence to support that adequate periodontal treatment generates an improvement in glycemic control in type 2 diabetic patients, evidenced by a reduction in glycated hemoglobin (HbA1c) levels between 0.29% and 0.48% that remains for at least three months after treatment. Although there is insufficient evidence on whether this reduction is maintained after six months of periodontal treatment [14,43,109,110,111]. This result has also been corroborated by other studies with a moderate quality of evidence [112,113,114,115,116,117,118] and by a Cochrane review [117]. The beneficial effect of periodontal treatment in diabetics also seems to translate into a reduction in inflammatory mediators evidenced through a reduction in serum levels of TNF-α and CRP [119,120]. However, it does not seem that the different types of periodontal treatment (surgical, non-surgical, accompanied or not by antibiotics, antiseptics, or with oral hygiene instructions) exert different effects on glycemic control in patients with DM.

Finally, there is reasonable evidence that indicates that PD could increase the risk of developing diabetes, since HbA1c levels have been increased in people with PD without diabetes [107,108]. The joint consensus meeting between the International Diabetes Federation and the European Federation of Periodontology [43] analyzed six representative studies from USA, Japan, and Taiwan populations, (*n* = 77,716 patients) showing a greater probability of developing prediabetes and diabetes (hazard ratio [HR] = 1.19–1.33) in patients with PDs.

### 3.4. Dental Implants, Peri-Implantitis, and DM

As mentioned, one of the fundamental consequences of PD in patients with DM is the loss of teeth, which occurs more markedly in elderly patients [69], and thus, one of the more subtle effects of the DM, especially DM2, could be the decrease in quality of life associated with tooth loss and compromised mastication function [121]. Modern dentistry restores lost teeth essentially through dental implants, which has been a real revolution in this field. However, questions have arisen regarding the feasibility and safety of dental implants in the diabetic population. On this issue there is scant and sometimes confusing evidence on how poorly controlled DM affects the prevalence of peri-implant disease, a process equivalent to PD [122,123], which implies bone loss around the implant [124]. In addition, there is also no consistent evidence about whether in patients with DM there is a significantly greater loss of dental implants after their placement [125,126,127], although apparently there is a delay in osseointegration of the implants related to the poor glycemic control [50,124,128]. A recent systematic review and meta-analysis [129] has indicated that there were statistically significant differences between the groups of DM and non-DM with regard to marginal bone loss (*p* < 0.001), probing depth (*p* < 0.001), and bleeding around dental implants (*p* < 0.001), obtaining the non-DM group the lower complication rates. Finally, in some studies it has been suggested that poorly controlled DM constitutes a relative contraindication for implant therapy [69], although on the contrary, numerous studies support the use of dental implant therapy in diabetic patients even with poor control of the glycaemia [130,131,132,133].

## 4. Oral Candidiasis

The relationship between fungal infections, and in particular infection by species of the genus *Candida* sp., with DM has been widely studied [10,134,135,136]. It has been clearly established that diabetic patients have an increased susceptibility to fungal infections compared to non-diabetics [137,138]. These susceptibility requires predisposing factors that decisively alter the balance between the host and the yeasts, allowing the passage of *Candida* sp. from its usual commensal state to pathogen, causing infection.

Among the different types of fungal infections that can occur in diabetic patients, oral candidiasis [6] stands out due to its higher frequency and clinical consequences. Significantly higher rates of colonization of the oral mucosa by *Candida* sp. have been described in patients with DM1 (85%) and DM2 (68%) compared with non-diabetics (27%) [138]. One study revealed that 66% of the yeasts isolated from DM patients were *C. albicans* [139]. However, fungal colonization of the oral mucosa is not equivalent to infection, requiring some pathophysiological conditions and associated factors for the infection to finally occur [9,140,141,142,143,144]. These factors are firmly established in diabetic patients and are as follows: (a) Maintained hyperglycemia with increased levels of HbA1c and high levels of glucose in saliva favors the multiplication of *Candida* sp., the increase in the number of receptors available for *Candida* sp., decreased neutrophil activity and increased adherence of *Candida* sp. to the epithelial cells of the oral mucosa [145,146,147,148,149,150,151]; (b) the decrease in salivary pH favors the growth of *Candida* sp., the increase in phospholipase and extracellular acid protease levels and the increase in the levels of yeast adhesion to epithelial cells [152,153,154]; (c) in DM there is a diminished response of the tissue to the injury favoring the colonization of the oral mucosa by *Candida* sp. even in the absence of clinical manifestations [155,156]; (d) and finally, poor oral hygiene, advanced age, female gender and xerostomia are also factors that can appear in DM and have been shown to be associated with a greater tendency to develop fungal infections in diabetics [157,158,159,160].

The common clinical manifestations of oral candidiasis are the presence of extensive reddened areas (erythematous candidiasis) along the oral mucosa, which are generally associated with patchy lingual depapilation and commissural cheilitis. Diabetic patients may present also speudomembranous candidiasis characterized by the presence of whitish lumps, similar to milk or yogurt clots, on an erythematous mucosa. These lumps are easily dislodged when scraped off with gauze leaving an erythematous mucosa. Oral candidiasis is usually symptomatic, causing discomfort, burning, or frank pain. Examination of the oral mucosa usually reveals, together with the events described, an absence of salivation or thick and pasty saliva. Diabetic patients may also develop a type of candidiasis associated with the use of removable dental prostheses called prosthetic stomatitis. It is characterized by the appearance of a reddened area under the prosthesis resin, being the mucosa not covered by the prosthesis respected. This form of candidiasis is usually asymptomatic, although a degree of discomfort may also occasionally occur.

## 5. Oral Cancer and Oral Potentially Malignant Disorders

Oral cancer is a global oral health problem. The most recent data published by prestigious entities (Global cancer incidence, mortality and prevalence [GLOBOCAN] project, International Agency for Research on Cancer [IARC], World Health Organization [WHO]) indicate the appearance of 354,864 new cases and 177,384 patients death per year [161], and a five-year-mortality rate of 50% directly related to this tumor [11]. A fact of great concern is that mortality from oral cancer has not decreased substantially in recent years, despite the fact that the oral cavity is explored by multiple specialists (otolaryngologists, maxillofacial surgeons, dermatologists, dentists, and family doctors). A systematic review and meta-analysis recently published by our research group indicates that diabetic patients have a significantly higher prevalence and risk of developing oral cancer compared to the general population [12]. Worldwide studies on oral cancer incidence and prevalence [162] indicate a strong geographical predisposition for the development of oral cancer, with India and Southeast Asian countries showing the highest figures. This geographical distribution seems to depend on the high levels of tobacco consumption in these countries as this habit is the most relevant etiological factor for the development of oral cancer. However, in our meta-analysis, the subgroup analysis showed the increased risk of development of oral cancer in the diabetic population not dependent on the geographical area studied. In our opinion, and based on these results, the predisposition to the development of cancer in the diabetic population depends directly from conditions associated with DM.

The reasons for the increased development of oral cancer in diabetics are not well known, although clinical, biochemical, and molecular reasons have been proposed. Furthermore, oral cancer and DM share some epidemiological facts and etiological factors, among which are obesity, sedentary lifestyle, advanced age, and diet [163]. On the other hand, hyperinsulinemia due to insulin resistance, through the activation of EGF1R, gives rise to the upregulation of some pro-proliferative and antiapoptotic pathways that have also been documented activated in oral cancer in non-diabetics (PI3K-akt-mTor, MAPK [Ras-Raf-MEK-Erk], and Bcl-2) [164]. Upregulation of these pathways conclusively concludes with the upregulation the *CCND1* gene [165,166]. Our research group has recently pointed out that the upregulation of *CCND1* and the overexpression of its product (cyclin D1) play a determining role in the cascade of molecular events that occur in the malignant transformation of the oral epithelium [167,168]. Therefore, it could be hypothesized that the link between DM and the development of oral cancer is hyperinsulinemia and insulin resistance [12]. Furthermore, as previously mentioned in this paper, hyperglycemia by generating oxidative stress with the release of ROS could cause DNA damage [169]. Hyperglycemia could also be accompanied by an increase in glucose consumption by tumor cells, also known as Warburg effect. This is a well-known hallmark of cancer proposed by Hanahan and Weinberg [170], which seems to induce an increase in cell proliferation associated with an activation of GLUT-1 and GLUT-3, and EGF, EGFR, and PKC-α [171,172,173].

We have also documented in our research line an increase in oral cancer-related mortality 2.09 times higher in the diabetic population compared to the non-diabetic (95%CI = 1.36–3.22, *p* = 0.001). This fact, which has also been observed in other types of cancers (liver, pancreas, ovarian, colon, lung, bladder, and breast carcinomas [174,175]) could be due to the phenotype more aggressive—proliferative and invasive—that develops cancer in diabetics as well as the deterioration of the general health of the diabetic related with complications (kidney disease, ischemic disease, etc. [176]) as well as the limitations for surgical treatment linked to postoperative risks, together with higher postoperative mortality [177].

Our research group has also reported the increased risk of development of oral potentially malignant disorders (OPMD) experienced by diabetic patients compared to the general population [12]. OPMDs are a significant group of mucosal disorders that may precede the diagnosis of oral squamous cell carcinoma (OSCC) [13,178], among which are essentially oral leukoplakia [179,180], oral lichen planus (OLP) [181,182,183], proliferative verrucous leukoplakia [184,185,186], erythroplakia, and actinic cheilitis [187,188]. Patients diagnosed with OPMDs may have an increased susceptibility to develop cancer anywhere in their mouth during their lifetime [13]. Our previous meta-analysis has shown that oral leukoplakia occurs with a prevalence of 2.49% in the diabetic population (2490 per 100,000 patients with DM) being the risk of developing oral leukoplakia in a diabetic 4.34 times higher compared to the general population. (95%CI = 1.14–16.55, *p* = 0.03; 10 studies, 7440 patients). A recent study by the WHO collaborative group for the study of cancer and OPMD [179] has reported a risk of oral cancer in oral leukoplakia close to 9%, which indicates the concern of the diagnosis of leukoplakia in a diabetic patient. The risk of developing oral leukoplakia in diabetics does not depend on the geographical area, nor does it depend on tobacco consumption, which indicates that it is probably related to factors exclusively associated with diabetes.

Our results are also strong for OLP, another important and highly prevalent OPMD [189] and associated with considerable malignant transformation rates [181,182,183]. Patients with DM present a prevalence of OLP of 2.72% (2720 per 100,000) with a chance of developing OLP 1.87 times higher than in the non-diabetic (95%CI = 1.37–2.57, *p* < 0.001; 22 studies, 5830 patients) [12,190]. Nowadays, the premalignant character of OLP and its progression to cancer high rate have been clearly documented [181], so it is reasonable to hypothesize that in a considerable number of patients diabetics, the appearance of oral cancer may come from the malignant evolution of a previous OLP.

## 6. Other Oral Conditions Associated with DM

### 6.1. Dental Caries

The analyses on the prevalence of dental caries in the diabetic population present contradictory and conflicting results [14]. It would seem logical to think that diabetic population, as a consequence of a series of associated oral conditions (xerostomia, high levels of dental plaque, etc.) would be more predisposed to the development of dental caries [191]. A recent systematic review and meta-analysis [15] has reported DM1 patients having significantly higher caries prevalence compared to controls. Although no significant differences were found between DM2 and controls and between well-controlled and poorly controlled diabetics. On the contrary, a study [192] with a large sample (300 diabetics vs. 300 controls) reported a higher prevalence of dental caries in non-diabetics, which the authors attribute to the fact that perhaps the diet of patients with DM generally contains less fermentable carbohydrates and more protein [193]. Another analysis did not find differences in the prevalence of crown caries, although significant differences were found for the prevalence of root caries [194].

### 6.2. Burning Mouth Syndrome (BMS)

It is an atypical chronic pain essentially characterized by the presence of a burning sensation, stinging or frank pain that is located mainly on the tongue, lips and palate, although it can spread to any other location, without that there are recognizable mucosal lesions that may justify this condition. BMS usually appears in women over 30 years of age and is frequently associated with a history of various emotional disorders [195]. It is a common disease, with an estimated prevalence ranging from 0.7% to 4.6% of the general population [16]. BMS is a process with an impact on the patients quality life, although, despite its frequency and relevance, the pathogenesis is unknown to a large extent [196]. In general, there is an absence of epidemiological primary-level studies focused on the association between BMS and DM. Increased prevalence of BMS in patients with DM compared to healthy subjects has been reported [197,198], while others did not find any differences in prevalence of BMS [199]. A significant association between BMS and peripheral neuropathy has been reported in diabetic patients [17]. It could indicate that BMS in diabetic patients constitutes another manifestation of diabetic neuropathy, although this is an unconfirmed theory.

### 6.3. Salivary Secretion Alterations

Alterations in salivary secretion are generically called by the term “dry mouth”, which however refers to two different processes, the first related to an objective reduction of salivary flow due to salivary hypofunction, defined by an unstimulated whole saliva flow rate of <0.1 mL/min, collected for 5 to 15 min, or chewing-stimulated whole saliva flow rate of <0.7 mL/min, collected for 5 min [200]; and secondly, “dry mouth” can also refer to the subjective sensation of lack of saliva in the absence of flow disorders [18]. The prevalence of salivary hypofunction with decreased salivary flow is estimated to range widely from 1% to 65% of the general population [201].

Dry mouth is one of the most common complaints in diabetic patients. Numerous cross-sectional studies have reported decreased salivary flow from both DM1 [19,202,203,204,205,206,207] as in DM2 [19,205,208,209,210,211,212,213,214,215,216,217,218]. The pathophysiology of the lack of salivary flow in DM is partly unknown. It has been hypothesized that the parotid innervation involvement in the context of diabetic neuropathy could somehow be involved in the decrease of salivary flow in these patients, although the studies present contradictory results [19,202,219,220,221]. It should also be noted that the tricyclic antidepressant, frequently used in the treatment of this disorder associated with diabetic neuropathy, produce dry mouth [222]. Some studies have reported alterations in the structure of the salivary glands in patients with DM, including vacuolization or acinar atrophy [223,224]. Likewise, in patients with DM it is common to find asymptomatic parotid enlargement that has been interpreted as a compensatory mechanism for salivary hypofunction [225].

Hyperglycemia seems to be another of the mechanisms responsible for the lack of saliva in diabetics. Significant decreases in salivary flow have been shown in poorly controlled DM compared to those with good glycemic control [19,202,211,212]. In this sense, the overexpression of AGE and RAGE, secondary to hyperglycemia, has been increased in the lacrimal gland tissue in diabetic animal models and associated with dry eyes [226]. Although something equivalent has not been investigated in lacrimal glands, at least theoretically this mechanism could also be operating to salivary hypofunction. RAGE overexpression has been observed in the submaxillary gland of diabetic rats [227]. It is also known that polyuria and osmotic diuresis secondary to hyperglycemia frequently appear in DM, which can trigger dehydration and compensatory hyposalivation [228,229].

### 6.4. Taste Perception Alterations

Taste perception alterations, mainly hypogeusia, have been reported both in patients with DM1 and DM2, in a significantly higher proportion than in controls [192]. These alterations have also been related to the development of obesity [21] secondary to hyperphagia [20]. Although the alterations in taste perception at the moment are of unknown cause, it has been hypothesized that the disorders of diabetic neuropathy and salivary hypofunction could be in the background of these alterations [192].

### 6.5. Halitosis

Patients with diabetes are predisposed to halitosis [22], having been reported that approximately 25% of patients with diabetes mellitus suffer from halitosis [230]. The pathogenesis of this disorder is probably related to the frequent presentation of gingivitis, periodontitis, dental caries and xerostomia, which prevents adequate self-cleaning of the oral mucosa. In addition, some of the bacteria that are frequently isolated in the infections of diabetic patients are anaerobes that contribute to the production of volatile products that increase halitosis [23]. In this sense, under the background of periodontitis, bacterial putrefaction and the generation of volatile sulfur compounds could lead to sulfide compound odor [22,231]. On the other hand, under the background of xerostomia, Koshimune et al. [232] found higher concentrations of methyl mercaptan and hydrogen sulfide in patients with salivary secretion alterations. Another study found an association between halitosis and increased HbA1c levels among type 2 diabetic subjects [233]. It was hypothesized that this relationship could be related to the phenomenon of ketoacidosis associated with poorly controlled diabetes [233]. Further studies are needed to explain the nature of this association.

### 6.6. Delayed Wound Healing

A tendency towards delayed wound healing has been described, especially in patients with poor control of their diabetes in whom long-term complications occur [24]. Probably these long-term complications affect the small terminal vessels, damaging them [25], which produces an insufficient supply of cellular nutrients through the blood circulation, decreasing the inflammatory and antibacterial response [26]. Elevated HbA1c levels ≥ 6.5% significantly increase the risk of developing infections after dental interventions and complications of surgical wound healing. For this reason, it is advisable to obtain better control of glycosylated hemoglobin figures [234]. However, those pathological processes in which it is suspected that their presence is contributing to poor diabetes control, and in which surgical treatment is required, should not be delayed in order to achieve a better metabolic control of the disease [234]. In these cases, post-surgical wound care should be maximized and clinical considerations should be made on the convenience of using antibiotics in each specific case [234]. Regarding the type of antibiotic to be used in diabetic patients, the basic rules of antibiotherapy should be respected, i.e., cultures should be performed in these patients in order to select the most effective antibiotic [235]. If necessary, the administration of a broad-spectrum antibiotic should be initiated pending the results of the sensitivity study, and this should be maintained if the study demonstrates its efficacy [235].

Finally, DM is frequently related to other pathological processes, such as hypertension, that require drugs that could also cause decreased salivary flow [19].

## 7. Need for an Interdisciplinary Team in the Care of Diabetic Patients in Relation to Their Oral Health. Information to the Diabetic Patients about Their Oral Health

From the foregoing it is deduced the importance of oral health in diabetic patients and the reciprocal relationships that exist between good metabolic control of DM and oral health. From this derives the need to establish interdisciplinary teams in the management of diabetic patients, among which dentists should necessarily be. The information available in this regard indicates, however, that at least half of primary care physicians and diabetes specialists do not have adequate knowledge about the importance of oral health in general and about PD in particular in diabetic patients. Furthermore, those clinicians who claim to have knowledge on the subject do not transfer it to their clinical practice and only a third of the professionals refer their patients for a dental consultation [236]. In fact, some studies conclude that active collaboration between dentists, primary care physicians and diabetes specialists does not exist, and the referral of patients to share their care according to competencies is absent. In this way, diabetic patients in many cases are receiving neither the information nor the adequate treatment in relation to their oral health problems [237].

### 7.1. Attitude of Primary Care Physicians and Specialists Involved in the Management of Diabetic Patients in Relation to Their Oral Health Care

- Clinicians should discuss with diabetic patients the importance of oral health in their disease in relation to the influence it exerts on the metabolic control of the disease and on the reduction of the risk of developing some of the potential complications of DM. Likewise, diabetic patient should be advised to periodically go to the dental clinic for review their oral status [69];
- Clinicians should screen for the main oral conditions that occur in diabetes. This screening should include the evaluation of the periodontal status through simple questions about the existence of spontaneous gingival bleeding or during mastication and brushing, the appearance of mobility or displacement of teeth, the loss of teeth, the presence of halitosis, and the existence of suppuration or periodontal abscesses. Likewise, the presence of erythematous or pseudomembranous candidiasis should be evaluated both through the presence of its symptoms (itching or oral pain) and its signs (oral mucosa affected by extensive red areas and imprecise limits or white areas in the form of lumps that come off easily when scraped with gauze);
- Clinicians should screen the main OPMDs that appear in diabetic patients with a higher prevalence than in the general population (essentially oral leukoplakia and OLP), as well as oral cancer (delimited red areas, ulcers or overgrowths of the oral mucosa older than 15 days) [13,178];
- Clinicians should perform a scrutiny of salivary flow alterations, essentially questioning the patient about the presence of dry mouth symptoms and examining the oral mucosa (obvious absence of saliva or thick saliva, with a parchment-like appearance of the oral mucosa);
- Clinicians should refer diabetic patients to the dental office in the event of any oral health problem detected during control and follow-up visits;
- Clinicians should seek basic training in oral health that allows them to detect the presence of oral disorders that appear in diabetes.

### 7.2. Attitude of Dentists in the Management of Diabetic Patients in Relation to Their Oral Health Care

- Dentists should discuss with their patients the mutual influences between oral health and diabetes by seeking information on how diabetes can affect oral health [238,239,240,241,242,243];
- Dentists should promote lifestyle changes on the habits of diabetic patients in order to exert a favorable impact on their oral and general health;
- Dentists must promote attitudes aimed at obtaining the maximum efficiency of oral care in diabetics [43,244]:
  - The medical history should be meticulous and detailed;
  - Communication with primary care physicians and other specialists involved in the care of diabetics should be fluid;
  - The intraoral examination should be meticulous looking for the frequent oral alterations in diabetics, with special reference to the signs and symptoms of PD, oral candidiasis, dry mouth and the presence of OPMD and oral cancer.
- The dental treatment of diabetics should focus on the control of acute infection, offering a therapy plan that is as less complex as possible. Likewise, emergencies in the dental clinic (hyperglycemia, hypoglycemia) must be recognized early and adequately managed. Considerations should be given to which are the most appropriate times to perform dental treatments and what should be the optimal duration of appointments, planning the treatment according to difficulties. Deep anesthesia and good pain and stress control should be provided during treatment;
- Dentists should advise and promote the replacement of missing teeth, the restoration of decayed teeth, and the implementation of preventive oral health habits;
- The dentist must be aware of the existence of the growing number of diabetics in the world [245], many of whom are undiagnosed [246,247]. Dental clinics could act as linkers involved in diabetes screening. In this sense, the suspicion of diabetes in a dental patient should prompt the dentist to request a check of glucose levels in venous blood and in case of alteration, the referral of the patient to his primary care physician for study and treatment if necessary [248];
- Dentists should seek basic training on DM and its complications.

### 7.3. Information Diabetic Patients Should Receive about Their Oral Health

- Diabetic patients should be given information about their oral health and its relationship to diabetes;
- Diabetic patients should receive information from dentists on the higher prevalence of PD in DM and on the negative consequences this has for the metabolic control of diabetes and on the presentation of complications of diabetes;
- Diabetic patients should receive information from dentists on habits and lifestyle that prevent the development of oral complications of diabetes;
- Diabetic patients should know that they are at risk of developing oral candidiasis;
- Diabetic patients should know that they are at risk of developing oral cancer and OPMD, through accurate, evidence-based information;
- Diabetic patients should know that they could develop alterations in salivary flow with dryness related to their disease;
- Diabetic patients should know the importance of making regular visits to the dental clinic;
- Diabetic patients must make commitments to their oral care.

### 7.4. Practical Measures and Recommendations to Follow in a Routine Dental Care Session

- Prior to dental treatment, a comprehensive medical history should be performed, singularly recording the type of diabetes, complications, treatment, and control status [249];
- International consensus guidelines state HbA1c levels <6.5% as the main parameter to measure and confirm an appropriate metabolic control [250];
- Pre-prandial blood glucose levels ranging between 70 and 130 mg/dL and post-prandial blood glucose levels < 180 mg/dL also should be confirmed to ensure an adequate metabolic control [250];
- Although well-controlled DM patients could be treated similarly to non-diabetics, short appointments in the morning are preferably to reduce stress of patients [248,251];
- At the beginning of each appointment, the dentist should make sure that the diabetic patient has eaten (fasting must be imperatively avoided) and taken their medications as usual, to avoid a hypoglycemic episode [248,251].

## 8. Conclusions

Diabetic patients present a notable predisposition to the development of oral pathologies, among which PD stands out, which reaches a prevalence of 67.8%. DM patients have a special predisposition to the development of fungal infections, especially of the *Candida* sp. genus, with significantly higher rates of oral mucosa colonization by *Candida* sp. both in patients with DM1 (85%) and DM2 (68%) compared to non-diabetics (27%). A higher prevalence of oral cancer and OPMD in diabetics has been reported, including oral leukoplakia, with a prevalence of 2.49% in patients, and oral lichen planus with a prevalence of 2.72%. Dental caries, burning mouth syndrome, alterations in saliva secretion, altered taste perception, halitosis, and delayed wound healing are also conditions associated with DM. All these disorders generate important complications that notably worsen the already deteriorated health status of diabetic patients. The frequent involvement of the oral cavity in these patients requires an interdisciplinary approach to its management and adequate guidelines for informing patients about these aspects. It is also essential to increase the training of diabetes care providers as well as patients in relation to their oral health.

## Figures and Tables

**Table 1:** Oral manifestations that may be present in patients with Diabetes Mellitus.

Oral Manifestations	Description	References
*Periodontitis*	The concept of periodontitis refers to a chronic inflammatory process characterized by microbially-associated, host-mediated inflammation that results in loss of periodontal attachment (i.e., by loss of marginal periodontal ligament fibers, apical migration of the junctional epithelium, and apical spread of the bacterial biofilm along the root surface of teeth). Initially, bacterial biofilm formation begins gingival inflammation (i.e., dental-biofilm induced gingivitis) with periodontitis initiation and progression. Furthermore, a multifactorial origin influenced by additional risk factors, such as smoking, is now supported on the immunoinflammatory bases of periodontitis. The relationship between periodontitis and DM (i.e., high prevalence and magnitude of association) is based on a solid evidence level.	[7,8]
*Oral candidiasis*	Fungal infections, particularly by species of the genus *Candida* sp. Common clinical manifestations are the presence of extensive reddened areas (erythematous candidiasis) along the oral mucosa, generally associated with patchy lingual depapillation and commissural cheilitis. DM patients may present whitish lumps, similar to milk or yogurt clots (speudomembranous candidiasis). Oral candidiasis is usually symptomatic, causing discomfort, burning, or frank pain.	[9,10]
*Oral cancer*	Oral cancer is the malignant neoplasm affecting lips, oral cavity, or oropharynx. Oral squamous cell carcinoma represents around 90% cases and has a 5-year mortality rate of 50%. The reasons for the increased development of oral cancer in diabetics are not well known, although clinical, biochemical, and molecular reasons have been proposed.	[11,12]
*Oral potentially malignant disorders (OPMD)*	OPMDs are a significant group of mucosal disorders that may have an increased susceptibility to develop oral cancer, which are essentially oral leukoplakia, oral lichen planus (OLP), proliferative verrucous leukoplakia, erythroplakia and actinic cheilitis. Leukoplakia and OLP—both prevalent OPMDs, associated with considerable malignant transformation rates—have a higher prevalence in subjects with DM than in general population.	[12,13]
*Dental caries*	Dental caries also known as tooth decays are caused by a breakdown of the tooth tissues. This breakdown is the result of dental plaque’s bacterias on teeth that produce acid destroying tooth tissues (e.g., enamel) and results in tooth decay. Diabetic patients, as a consequence of a series of associated oral conditions—xerostomia, high levels of dental plaque, etc.—would be more predisposed to the development of dental caries.	[14,15]
*Burning mouth syndrome (BMS)*	BMS is an atypical chronic pain essentially characterized by the presence of a burning sensation, stinging, or frank pain that is located mainly on the tongue, lips, and palate, although it can spread to any other location, without that there are recognizable mucosal lesions that may justify this condition. BMS seems to present an increased prevalence in patients with DM compared to healthy subjects. It could be associated with the peripheral neuropathy frequently reported in diabetic patients.	[16,17]
*Salivary secretion alterations*	Alterations in salivary secretion are generically known by the term “dry mouth”, which however refers to two different processes, the first related to an objective reduction of salivary flow due to salivary hypofunction (i.e., unstimulated whole saliva flow rate of <0.1 mL/min); and the second, the subjective sensation of lack of saliva in the absence of flow disorders. Dry mouth is one of the most common complaints in diabetic patients, with a partially unknown pathophysiology, which could be related to diabetic neuropathy of parotid gland, pathologic alterations in the salivary glands structure (e.g., vacuolization or acinar atrophy), or hyperglycemia and poorly controlled DM.	[18,19]
*Taste perception alterations*	Taste perception alterations, mainly hypogeusia (i.e., a partial loss of taste) have been reported in patients with DM. The reasons for the increased development of taste perception alterations in diabetics are not well known, although it has been proposed that it could be associated with the peripheral neuropathy frequently reported in diabetic patients.	[20,21]
*Halitosis*	Halitosis is a symptom where a person has bad breath. It can be caused by bad oral health, singularly dental care, caries, or periodontitis. Patients with DM are predisposed to halitosis, probably related to the frequent prevalence of these diseases in diabetic patients.	[22,23]
*Delayed wound healing*	Delayed wound healing is a complication in diabetics after oral surgery, especially in patients with poorly controlled DM. The probable cause of delayed wound healing is damaged small terminal vessels, responsible of reduced blood flow, with an insufficient supply of cellular nutrients through the blood circulation, decreased inflammatory and immune response.	[24,25,26]
